# Assessment of Variation in Cesarean Delivery Rates Between Public and Private Health Facilities in India From 2005 to 2016

**DOI:** 10.1001/jamanetworkopen.2020.15022

**Published:** 2020-08-28

**Authors:** Mrigesh Bhatia, Kajori Banerjee, Priyanka Dixit, Laxmi Kant Dwivedi

**Affiliations:** 1 Department of Health Policy, London School of Economics, London, United Kingdom; 2 Department of Mathematical Demography and Statistics, International Institute for Population Sciences, Mumbai, India; 3 Centre for Health and Social Sciences, Tata Institute of Social Sciences School of Health Systems Studies, Mumbai, India

## Abstract

**Importance:**

The rates of cesarean deliveries have more than doubled in India, from 8% of deliveries in 2005 to 17% of deliveries in 2016. The World Health Organization recommends that cesarean deliveries should not exceed 10% to 15% of all deliveries in any country. An understanding of the association of private and public facilities with the increase in cesarean delivery rates in India is needed.

**Objective:**

To assess the association of public vs private sector health care facilities with cesarean delivery rates in India and to estimate the potential cost savings if private sector facilities followed World Health Organization recommendation for cesarean deliveries.

**Design, Setting, and Participants:**

This cross-sectional study used institutional delivery data from the representative National Family Health Survey (NFHS) in India, including data from the NFHS-1 (1992-1993), the NFHS-3 (2005-2006), and the NFHS-4 (2015-2016). The NFHS-3 and NFHS-4 provided data on 22 647 deliveries and 195 366 deliveries, respectively. The NHFS-4 was the first survey to provide data on out-of-pocket expenditures for delivery by facility type, allowing for a comparison of cesarean deliveries and costs between public and private facilities. The primary sample comprised all pregnant women who delivered infants in public and private institutional facilities in India and who were included the NFHS-3 and the NFHS-4; data on pregnant women who were included in the NFHS-1 were used for comparison. The study’s findings were analyzed through geographic mapping, data tabulation, funnel plots, multivariate logistic regression analyses, and potential cost-savings scenario analyses. Data were analyzed from June to December 2019.

**Main Outcomes and Measures:**

The main outcome was the rate of cesarean deliveries by facility type (public vs private) and by participant socioeconomic, demographic, and health characteristics. Secondary outcomes were the potential number of avoidable cesarean deliveries and the potential cost savings if private sector facilities followed the World Health Organization recommendations for cesarean deliveries.

**Results:**

In the NFHS-3, 22 610 total births occurred at institutional facilities. Of those, 2178 births (15.2%) were cesarean deliveries in public facilities, and 3200 births (27.9%) were cesarean deliveries in private facilities. Of 195 366 total institutional births in the NFHS-4, 15 165 births (11.9%) were cesarean deliveries in public facilities, and 20 506 births (40.9%) were cesarean deliveries in private facilities. The cesarean delivery rate in public health facilities increased from 7.2% in the NFHS-1 to 11.9% in the NFHS-4, whereas in private health facilities, the rate increased from 12.3% to 40.9% during the same period. A substantial increase was found in cesarean delivery rates between the NFHS-3 (2005-2006) and the NFHS-4 (2015-2016), with 22 states exceeding the World Health Organization’s upper threshold of 15% in the NFHS-4. The odds ratio for cesarean deliveries in private facilities compared with public facilities increased from 1.62 (95% CI, 1.49-1.76) in the NFHS-3 to 4.17 (95% CI, 4.04-4.30) in the NFHS-4. The number of avoidable cesarean deliveries would have been 1.83 million, with a potential cost savings of $320.60 million, if private sector facilities in India had followed the 15% threshold for cesarean delivery rates recommended by the World Health Organization.

**Conclusions and Relevance:**

In this study, private sector health facilities were associated with a substantial increase in cesarean deliveries in India. Further research is needed to assess the factors underlying the increase in cesarean deliveries in private sector facilities.

## Key Points

**Question:**

Are private vs public sector health care facilities associated with increases in cesarean delivery rates among pregnant women in India over time, and what is the avoidable burden of cesarean deliveries in private sector facilities?

**Findings:**

In this cross-sectional study of 217 976 births at public and private sector institutions in India between 2005 and 2016, the likelihood of having a cesarean delivery in a private facility more than doubled over the period examined. A reduction in the percentage of cesarean deliveries in the private sector to the World Health Organization’s recommended threshold of 15% was associated with a potential cost savings of approximately $321 million.

**Meaning:**

The study’s findings indicated that private sector facilities were associated with increases in the rate of cesarean deliveries; it is important that policy makers address the increasing number of avoidable cesarean deliveries in India.

## Teaser

This cross-sectional study uses data from the National Family Health Survey to assess the association of public vs private sector health facilities with the increase in cesarean delivery rates and to estimate the avoidable burden of cesarean deliveries in India between 2005 and 2016.

## Introduction

 In the past several decades, a pattern of rapid increases in cesarean delivery rates has been observed worldwide, and this increase has varied across regions.^1^ Although these rates have increased at a slow pace in countries within sub-Saharan Africa,^2^ they are increasing at a substantial rate in many other countries. For instance, in the US, the cesarean delivery rate reached 30% in 2006, partly owing to the practice of preventive medicine and the threat of litigation.^3^ In European countries, the cesarean delivery rates vary from 52.2% in Cyprus to 14.8% in Iceland, with rates in the United Kingdom ranging from 24.6% in England to 29.9% in Northern Ireland.^4^ Australia’s cesarean delivery rate increased from less than 20% in 1998 to approximately 30% in 2008.^5^ Moreover, in Asia, an increase in cesarean delivery rates has been observed in a number of countries, including India, Nepal, China, and Bangladesh.^6^ Such a substantial increase in cesarean delivery rates without an indication of benefits for maternal or neonatal health has become a major public health concern.^7^

Although cesarean delivery can be a life-saving surgery, this procedure should be performed only when medically indicated, as complications that have adverse consequences for the mortality and morbidity of both the mother and the newborn are well documented in the literature.^8,9,10,11,12,13,14,15,16^ Some of the negative health outcomes in infants born via cesarean delivery include childhood obesity, respiratory disorders, type 1 diabetes, acute lymphoblastic leukemia, impaired cognitive development, higher rates of autism, and an increased risk of neurodevelopmental disorders.^15,17,18,19,20,21,22,23^ Cesarean delivery has been reported to be associated with an approximately 4-fold increase in the risk of maternal death.^24^ In addition, unnecessary cesarean deliveries may be associated with higher health care costs in many low-income settings.^25^

India has also experienced increases in cesarean delivery rates similar to those observed in the rest of the world. Based on our calculations, cesarean delivery rates have more than doubled in India as a whole, from 8% in 2005 through 2006 to 17% in 2015 through 2016. The World Health Organization (WHO) recommends that the percentage of cesarean deliveries should not exceed 10% to 15% in any nation. The present study assessed the variation in cesarean delivery rates in public and private sector health facilities in India to evaluate whether private facilities were associated with increases in cesarean delivery rates and to estimate the burden of avoidable cesarean deliveries in the private sector.

## Methods

The data for this cross-sectional study were obtained from the National Family Health Survey (NFHS), which is a nationally representative survey conducted under the stewardship of the Ministry of Health and Family Welfare in India. The International Institute for Population Sciences in Mumbai is designated as the central agency to implement the survey. Although our analysis was mainly based on data from the most current survey, the NFHS-4 (2015-2016),^26^ data from previous rounds of surveys, specifically the NFHS-1 (1992-1993)^27^ and the NFHS-3 (2005-2006),^28^ were also used. The present study calculated the patterns in cesarean delivery rates in India by type of facility and assessed the association of participants’ sociodemographic, economic, and health characteristics and their place of delivery with the likelihood of having a cesarean delivery. The study also examined the potential cost savings of reducing the current cesarean delivery rates in the private sector to the thresholds recommended by the WHO. This study followed the Strengthening the Reporting of Observational Studies in Epidemiology (STROBE) reporting guideline for cross-sectional studies. Ethical review was not necessary as this study was based on the analysis of secondary survey data, which is available in the public domain, and complied with all requirements of 45 CFR §46.

For comparison, the locations of institutional deliveries were classified into public and private sectors. A dichotomous variable was created based on the location of the live birth. The location was considered public if the delivery occurred in a government hospital, government dispensary, urban health center, urban family welfare clinic, community health center, or primary health center. The location was considered private if the delivery occurred at a private hospital or private clinic. Owing to their small numbers, nongovernmental organizations and trust hospitals were also included in the private sector category.

### Statistical Analysis

Geographic maps were developed to visualize the change in spatial distribution of the cesarean delivery rate in India from the NFHS-3 to the NFHS-4. A total of 7 cutoffs for the percentage of cesarean deliveries (3 cutoffs lower and 4 cutoffs higher than the 15% threshold recommended by the WHO) were used to highlight the increase in cesarean delivery rates in various states and union territories of India. In addition, funnel plots were drawn to observe the variation in the percentage of cesarean deliveries according to public vs private facilities in the states. We constructed 95% CI bands in funnel plots to identify states with rates higher than the 95% CI band, which were considered upper outliers with high cesarean delivery rates, and states with rates lower than the 95% CI band, which were considered lower outliers with low cesarean delivery rates. The funnel was plotted using the lower and upper control limits, which were calculated from the aggregated national cesarean delivery percentages and SEs based on state rates.

A multivariate binary logistic regression model was constructed to estimate the increase in the likelihood of cesarean delivery in private vs public health facilities. Cesarean delivery was a binary variable coded as 0 for a vaginal delivery and 1 for a cesarean delivery; thus, an odds ratio (OR) greater than 1 signified that the OR of a cesarean delivery for that particular explanatory variable was higher than that of the reference category. We considered several relevant background characteristics and assessed their associations with the likelihood of having a cesarean delivery. The explanatory variables considered were the size of the child at birth (small, average, or large, as reported by the mother), birth order of the child (1, 2 or ≥3), maternal age at the child’s birth (≤19 years, 20-29 years, or ≥30 years), maternal body mass index (BMI; calculated as weight in kilograms divided by height in meters squared; underweight [BMI, <18.49], normal [BMI, 18.50-24.9], and overweight or obese [BMI, ≥25]), maternal educational attainment (no formal education, primary school, secondary school, or higher education [≥12 years]), household wealth quintile (defined by the NFHS wealth index as the relative index of household wealth based on the standard set of assets owned by the household, including ownership of consumer items and dwelling characteristics; 5 categories of wealth quintiles [poorest, poorer, middle, richer, and richest] from the NFHS wealth index were used), household caste (scheduled caste or scheduled tribe, other backward class, or other caste), household religion (Hindu or non-Hindu), area of residence (urban or rural), and health care facility type (public or private).

Data on institutional births were pooled to understand the association of time with the probability of cesarean delivery. The pooled model was adjusted for all of the control variables along with time and the interaction terms between time and selected explanatory variables. The interaction terms were included in the model to adjust for any time-varying effects of the independent variables. The adjusted percentages of cesarean delivery rates for selected background variables were calculated from this model.

To understand whether there were fundamental differences with respect to medical indications for cesarean delivery among women in public vs private facilities, a simple bivariate analysis was performed. The medical indicators considered were complications during pregnancy, complications during delivery, and preplanned cesarean delivery.

A scenario analysis was performed to estimate the economic burden of avoidable cesarean deliveries in the private sector by calculating the cesarean deliveries that could have been avoided and the potential cost savings that could have been achieved under various scenarios. Data regarding household out-of-pocket expenditures for vaginal and cesarean deliveries, which were available for the first time in the NFHS-4, were used for this analysis. All analyses were performed using Stata software, version 13.1 (StataCorp). Data were analyzed from June to December 2019.

## Results

The analysis considered only deliveries that occurred at institutional facilities. In the NFHS-3, 22 610 total births occurred at institutional facilities. Of those, 2178 births (15.2%) were cesarean deliveries in public facilities, and 3200 births (27.9%) were cesarean deliveries in private facilities. Of 195 366 total institutional births in the NFHS-4, 15 165 births (11.9%) were cesarean deliveries in public facilities, and 20 506 births (40.9%) were cesarean deliveries in private facilities.

**Figure 1.**
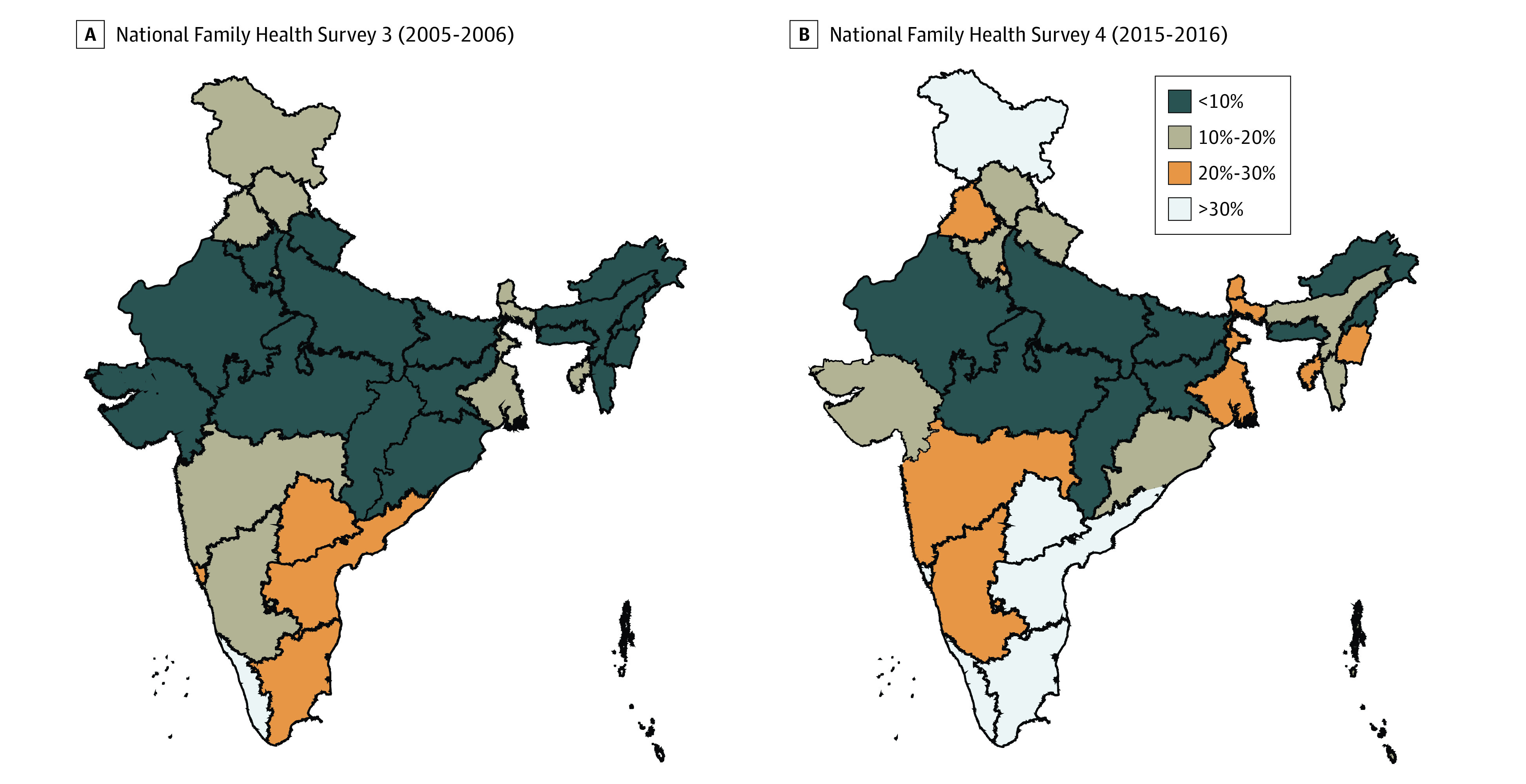
Change in Percentage of Cesarean Deliveries in India Cesarean delivery rates by state and/or union territory.

The rate of cesarean deliveries increased almost 7-fold from the NFHS-1 (1992-1993) to the NFHS-4 (2015-2016). Over 10 years, from the NFHS-3 to the NFHS-4, the overall rate of cesarean deliveries increased from 8.5% to 17.2%. The cesarean delivery rate in public health care facilities increased from 7.2% in the NFHS-1 to 11.9% in the NFHS-4. In private health care facilities, the rate increased 3-fold, from 12.3% in the NFHS-1 to 40.9% in the NFHS-4 (eTable in the Supplement). All of the states and union territories in India experienced a substantial increase in cesarean delivery rates over 20 years. A total of 6 states in the NFHS-3 and 22 states and union territories in the NFHS-4 exceeded the recommended WHO thresholds of 10% to 15% (Figure 1).

Over the last NFHS decade examined (2005-2006 through 2015-2016), a substantial increase in births at institutional facilities in India occurred. In the NFHS-3, 38.8% of deliveries occurred in a health care facility; this estimate doubled to 79.0% of deliveries in the NFHS-4. Although the increase in the cesarean delivery rates may be interpreted as a manifestation of the overall increase in institutional births, when only institutional deliveries were considered, the proportion of cesarean deliveries also substantially increased, specifically in the private sector. In the NFHS-3, the percentage of cesarean deliveries in private institutions was 27.9%, which increased to 40.9% in the NFHS-4. In contrast, the percentage of cesarean deliveries in public institutions decreased from 15.2% in the NFHS-3 to 11.9% in the NFHS-4 (eFigure 1 in the Supplement).

Funnel plots for cesarean delivery rates in private facilities from the NFHS-3 and the NFHS-4 are available in eFigure 2 and eFigure 3 in the Supplement. These figures identify the outlier states and the shift from the NFHS-3 to the NFHS-4. The dotted lines define the upper and lower 95% CI boundaries. States with cesarean delivery rates beyond these boundaries were considered outliers. In the NFHS-3, only 2 states, Andhra Pradesh and West Bengal, were observed to be upper outliers with high cesarean delivery rates; in the NFHS-4, the number of upper outliers increased to 13 states.

Table 1 provides the ORs of cesarean deliveries, which were controlled for various background characteristics in the NFHS-3 and the NFHS-4 separately. After controlling for maternal socioeconomic characteristics, educational levels, BMIs, and area of residence, the ORs of cesarean deliveries were found to be uniformly higher in private facilities compared with public facilities for both survey rounds. The results from the multivariate logistic regression analysis revealed that the OR of a cesarean delivery occurring in a private health care facility was 1.62 (95% CI, 1.49-1.76) in the NFHS-3 and 4.17 (95% CI, 4.04-4.30) in the NFHS-4 (*P* < .001). Health variables, such as older maternal age, higher BMI, and larger size of the child at birth had positive associations with the likelihood of having a cesarean delivery. For example, the OR of a cesarean delivery among women 30 years and older was 2.44 (95% CI, 2.07-2.88) in the NFHS-3 and 2.26 (95% CI, 2.11-2.43) in the NFHS-4 compared with an OR of 1.43 (95% CI, 1.25-1.63) in the NFHS-3 and 1.42 (95% CI, 1.34-1.50) in the NFHS-4 among women aged 20 to 29 years. Women who had higher educational levels and households in the richest wealth quintile also had a significantly higher likelihood of having a cesarean delivery in both survey rounds. Among women with a primary school education, the OR for cesarean delivery was 1.19 (95% CI, 1.02-1.37) in the NFHS-3 and 1.20 (95% CI, 1.14-1.27) in the NFHS-4. In comparison, women with higher education (≥12 years) had an OR of 1.56 (95% CI, 1.34-1.81) in the NFHS-3 and 1.35 (95% CI, 1.28-1.43) in the NFHS-4. For women in the poorer wealth quintile, the OR for cesarean delivery was 1.16 (95% CI, 0.92-1.47) in the NFHS-3 and 1.20 (95% CI, 1.14-1.27) in the NFHS-4. In comparison, women in the richest wealth quintile had an OR of 1.50 (95% CI, 1.19-1.89) in the NFHS-3 and 1.86 (95% CI, 1.74-1.99) in the NFHS-4.

**Table 1.** Logistic Regression Analysis of Cesarean Deliveries^**a**^

Variable	Odds ratio (95% CI)	
	NFHS-3 (2005-2006)	NFHS-4 (2015-2016)
Size of child^b^		
Average	1 [Reference]	1 [Reference]
Large	1.13 (1.04-1.23)	1.19 (1.15-1.23)
Small	1.09 (0.99-1.20)	1.12 (1.07-1.17)
Birth order of child		
1	1 [Reference]	1 [Reference]
2	0.73 (0.67-0.79)	0.76 (0.73-0.78)
≥3	0.40 (0.35-0.44)	0.39 (0.38-0.41)
Maternal age at birth, y		
≤19	1 [Reference]	1 [Reference]
20-29	1.43 (1.25-1.63)	1.42 (1.34-1.50)
≥30	2.44 (2.07-2.88)	2.26 (2.11-2.43)
Maternal BMI^c^		
Underweight	1 [Reference]	1 [Reference]
Normal	1.33 (1.21-1.46)	1.24 (1.20-1.29)
Overweight or obese	2.15 (1.91-2.41)	2.26 (2.16-2.37)
Maternal educational level		
No formal education	1 [Reference]	1 [Reference]
Primary school	1.19 (1.02-1.37)	1.20 (1.14-1.27)
Secondary school	1.19 (1.05-1.34)	1.29 (1.24-1.35)
Higher education (≥12 y)	1.56 (1.34-1.81)	1.35 (1.28-1.43)
Place of delivery		
Public facility	1 [Reference]	1 [Reference]
Private facility	1.62 (1.49-1.76)	4.17 (4.04-4.30)
Household wealth quintile^d^		
Poorest	1 [Reference]	1 [Reference]
Poorer	1.16 (0.92-1.47)	1.20 (1.14-1.27)
Middle	1.22 (0.98-1.52)	1.58 (1.50-1.68)
Richer	1.38 (1.10-1.72)	1.74 (1.64-1.85)
Richest	1.50 (1.19-1.89)	1.86 (1.74-1.99)
Area of residence		
Urban	1 [Reference]	1 [Reference]
Rural	0.98 (0.90-1.07)	0.87 (0.85-0.90)
Caste^e^		
Scheduled caste or scheduled tribe	1 [Reference]	1 [Reference]
Other backward class	0.96 (0.86-1.07)	1.00 (0.96-1.03)
Other castes	1.02 (0.92-1.13)	1.18 (1.14-1.23)
Religion		
Hindu	1 [Reference]	1 [Reference]
Non-Hindu	0.91 (0.83-1.00)	0.91 (0.88-0.95)
Constant^f^	0.1 (0.1-0.1)	0

Abbreviations: BMI, body mass index (calculated as weight in kilograms divided by height in meters squared); NFHS, National Family Health Survey.

^a^ Model was also fitted for all major states that were commonly included in both the NFHS-3 and the NFHS-4 for comparability.

^b^ Size of child at birth as reported by the mother.

^c^ Underweight was defined as a BMI of less than 18.49, normal weight as a BMI of 18.50 to 24.99, and overweight or obese as a BMI of 25.00 or higher.

^d^ Defined by the NFHS wealth index as the relative index of household wealth based on the standard set of assets owned by the household, including ownership of consumer items and dwelling characteristics. Five categories of wealth quintiles (poorest, poorer, middle, richer, and richest) from the NFHS wealth index were used.

^e^ Caste system categories as defined by the government of India.

^f^ Constant is the intercept of the logistic model that provides the log of the odds of C-section when all other variables are set to the reference category.

**Figure 2.**
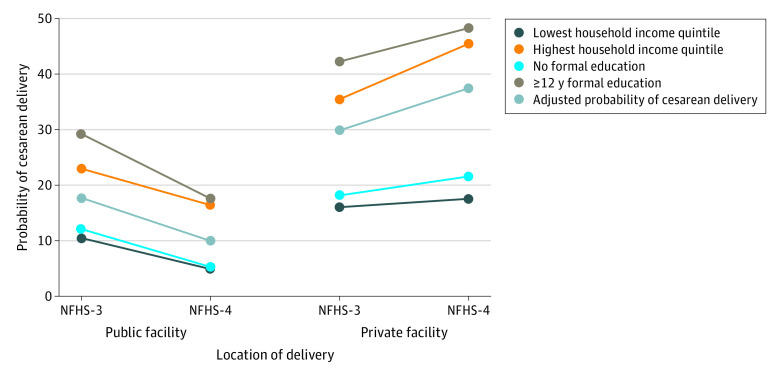
Adjusted Probabilities of Cesarean Delivery by Household Wealth Quintile and Maternal Educational Level Based on place of delivery from pooled logistic regression analysis of National Family Health Survey 3 (NFHS-3; 2005-2006) and National Family Health Survey 4 (NFHS-4; 2015-2016). Probabilities are reported as percentages.

The 2 rounds of the NFHS were also pooled to assess the association of socioeconomic status with the likelihood of cesarean delivery by the place of delivery. Figure 2 presents the adjusted probabilities of cesarean delivery by household wealth quintile and maternal educational level along with the adjusted estimates for the place of delivery from the pooled logistic regression analysis for institutional births only. Household wealth quintile and maternal educational level were 2 of the most important factors associated with cesarean delivery. For example, in private facilities, the probability of cesarean delivery among women in the poorest quintile was 16.0% in the NFHS-3 and 17.5% in the NFHS-4. In comparison, the probability of cesarean delivery among women in the richest wealth quintile was 35.4% in the NFHS-3 and 45.3% in the NFHS-4. Among women with no formal education, the probability of cesarean delivery at a private facility was 18.1% in the NFHS-3 and 21.5% in the NFHS-4. However, women with 12 years or more of formal education had a probability of cesarean delivery of 42.1% in the NFHS-3 and 48.1% in the NFHS-4. After pooling the data over time, we observed that the adjusted probability of cesarean deliveries in private facilities increased from 29.8% to 37.3%.

**Figure 3.**
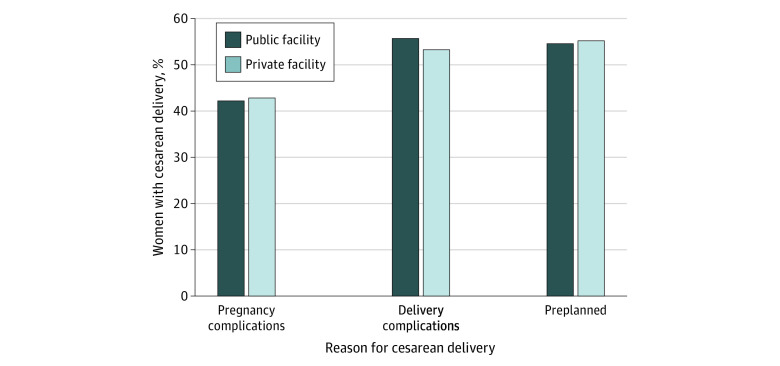
Percentage of Women With Cesarean Deliveries in India, 2015 to 2016.

The likelihood of cesarean delivery in private sector facilities in the NFHS-4 was not associated with medical indications for cesarean delivery with respect to delivery-associated complications, pregnancy-associated complications, or the decision to have a cesarean delivery before the onset of labor. No significant difference was found between these indicators among women who visited public vs private facilities; among women who had cesarean deliveries in public vs private facilities, 42.2% of women vs 42.9% of women had pregnancy complications, 55.7% of women vs 53.3% of women had delivery complications, and 54.6% of women vs 55.2% of women had preplanned cesarean deliveries, respectively (Figure 3).

Table 2 presents the results of a scenario analysis that compared the actual cesarean delivery rates in the public and private sectors with the hypothetical cesarean delivery rates if India had followed the WHO thresholds of 10% and 15%. Assuming the private sector experienced the mean national cesarean delivery rate, the potential number of avoidable cesarean deliveries would be 1.67 million, with a potential cost savings of $293.36 million. The avoidable cesarean deliveries and potential cost savings would be larger if the WHO cutoff rates of 10% and 15% were followed. At the 10% threshold, the potential number of avoidable cesarean deliveries would be 2.18 million, with a potential cost savings of $382.49 million. At the 15% threshold, the potential number of avoidable cesarean deliveries would be 1.83 million, with a potential cost savings of $320.60 million.

**Table 2.** Scenario Analysis of Potential Number of Avoidable Cesarean Deliveries in Private Sector Facilities and Potential Cost Savings, 2015 to 2016^**a**^

Scenario	Rate of cesarean deliveries, %	Total cesarean deliveries in private facilities, No., millions^b^^,^^c^	Avoidable cesarean deliveries, No., millions^d^	Total costs of cesarean deliveries in private facilities, $, millions^e^^,^^f^	Avoidable costs of cesarean deliveries, $, millions^g^	Potential cost savings, $, millions^h^
Private sector rate	40.9	2.89	NA	932.11	NA	NA
Mean national rate	17.2	1.22	1.67	391.99	540.12	293.36
Public sector rate	11.9	0.84	2.05	271.20	660.91	358.97
WHO lower threshold of 10%	10.0	0.71	2.18	227.90	704.21	382.49
WHO higher threshold of 15%	15.0	1.06	1.83	341.85	590.26	320.60

Abbreviation: NA, not applicable (indicates actual numbers for original private sector rates of cesarean delivery).

^a^ Avoidable cesarean deliveries, costs, and potential savings under alternate scenarios were estimated as the differences from the estimates at the original rate of cesarean deliveries in private facilities, which was 40.9%.

^b^ Total cesarean deliveries in 2015 to 2016 were obtained from the Sample Registration System database. Estimates from 2016 were based on a cesarean delivery rate of 20.4% and the total midyear population projected in Table 18 of the *Census of India 2011*.^45^

^c^ Total cesarean deliveries in the private sector were calculated as the total number of estimated deliveries multiplied by the proportion of all deliveries that occurred in the private sector multiplied by the proportion of private-sector deliveries that were cesarean deliveries.

^d^ Avoidable cesarean deliveries were calculated as the total number of caesarean deliveries in private facilities per the original rate of 40.9% minus the total number of cesarean deliveries that would have occurred if the rate of cesarean deliveries in private facilities had been reduced to the alternative scenarios.

^e^ The mean costs of cesarean deliveries in the private sector were $322.6 million, and the mean costs of vaginal deliveries in the private sector were $147.4 million, at a conversion rate of $1.00 to 75.326 Indian rupees (as of June 3, 2020).^46^

^f^ The total costs of caesarean deliveries were calculated as the total number of cesarean deliveries multiplied by the mean costs of cesarean deliveries in private facilities.

^g^ The avoidable costs of cesarean deliveries were calculated as the total costs in private facilities per the original rate of 40.9% minus the total costs that would have occurred if the rate of cesarean deliveries in private facilities had been reduced to the alternative scenarios.

^h^ The potential cost savings was calculated after adjusting for excess cesarean deliveries by translating them into vaginal deliveries, as follows: total costs for cesarean deliveries in private facilities per the original rate of 40.9% minus total costs for cesarean deliveries in private facilities per the alternative scenarios plus total costs for vaginal deliveries for the difference in deliveries owing to the original rate and the alternative rate in private facilities.

## Discussion

From 2005 to 2016, the rate of cesarean delivery increased from 8.5% to 17.2% in India. The cesarean delivery rate in public facilities increased from 7.2% in the NFHS-1 to 11.9% in the NFHS-4, whereas in private health care facilities, the rate increased from 12.3% to 40.9% during the same period, indicating a substantial distributional gap in cesarean deliveries between the public and private sectors. Consistent with our findings, other studies have reported higher rates of cesarean deliveries in the private sector.^29,30^

Our study also found that the likelihood of having a cesarean delivery in the private sector was higher (OR, 1.62 in the NFHS-3; OR, 4.17 in the NFHS-4) than in the public sector. In addition, the difference in the probability of having a cesarean delivery in public vs private sector facilities in both rural and urban India has increased over time. Older women, women with a higher BMI and educational level, and women belonging to wealthier households had a statistically greater likelihood of having a cesarean delivery. In addition, the adjusted probabilities of cesarean delivery remained high in private health facilities regardless of any change in family socioeconomic status over the decade between the NFHS-3 and the NFHS-4. A number of other studies have found positive associations of cesarean delivery with richer wealth quintiles^31,32^ and higher educational levels.^33^ In addition, other studies’ results are consistent with our finding that the place of delivery (ie, public vs private facility) is the most important structural factor in the outcome of birth by vaginal or cesarean delivery.^16,34^

Our results also highlight the fact that there were no substantial differences in medical indications for cesarean delivery (eg, pregnancy complications, delivery complications, or the decision to have cesarean delivery before the onset of labor) among women at public vs private facilities. Therefore, other nonmedical factors are likely to play a more substantial role in the increase of cesarean delivery rates in the private sector.

One of the factors documented in the literature that is associated with the increase in cesarean delivery rates is the role of private sector facilities in a number of settings. In many low- and middle-income countries, the introduction of health sector reforms has involved engagement with the private sector in the form of public-private partnerships. A number of such approaches have been successful in addressing the issue of safe motherhood in low- and middle-income countries.^35,36^ In India, the private sector has expanded rapidly, and government-sponsored health care programs rely on private hospitals as part of public–private partnerships.^37^

In this context, it is important to understand the characteristics of the private sector in India, which provides a range of health care services in both urban and rural areas.^38^ Private-sector hospitals range from small family-run general hospitals to facilities providing superspecialty tertiary care. Consultation fees vary because there is no fixed fee schedule, and patients usually pay for services directly (ie, out of pocket).^38^ In addition, the private health care sector in India is not well regulated,^37^ and millions of Indians experience impoverishment every year owing to high health care costs.

Our study also estimated that, assuming the private sector experienced the mean national cesarean delivery rate, the potential number of avoidable cesarean deliveries would be 1.67 million, with a potential cost savings of $293.36 million. Consistent with our findings, 1 study estimated a potential 0.9 million preventable cesarean deliveries in the private sector in India.^39^ Another study estimated that 6.2 million unnecessary cesarean deliveries were performed globally in 2008 at a cost of $2.32 billion.^40^ Such avoidable cesarean deliveries consume a large share of national and global resources, have equity implications, and act as a barrier to achieving universal health coverage.^40^

### Limitations

This study has several limitations. Although cesarean delivery rates were estimated based on NFHS guidelines, all limitations for analyses of sample survey data also applied to our study. In addition, because the study used secondary data, it was not possible to capture the appropriateness of cesarean deliveries performed in public or private facilities. Exploring the underlying factors associated with high cesarean delivery rates in the private sector was beyond the scope of this study.

From our results, it appears that India is in the early stages of an increasing pattern of cesarean deliveries. As seen in the funnel plot, the number of highly populated states with high birth rates is behind the curve with respect to cesarean delivery rates. Therefore, the consequences of higher cesarean delivery rates in India will likely be more noticeable when highly populated states, such as Bihar and Uttar Pradesh, start to experience cesarean delivery rates similar to those in some of the less populated states, such as Andhra Pradesh, Tamil Nadu, Gujarat, and West Bengal. Hence, policy makers in India have a window of opportunity to forestall the increase in cesarean deliveries before it occurs in highly populated states.

A number of approaches can be considered by the government of India to address the problem of high cesarean delivery rates in private sector facilities; these approaches include informing patients of the risks of the cesarean delivery procedure, including the higher probability of subsequent births by cesarean delivery. In Brazil, it is now mandatory for pregnant women to acknowledge the risks of a cesarean delivery before surgery, and this requirement has inspired partnerships with several hospitals to promote vaginal birth.^41^ Through public-private partnerships, the government of India could use financial incentives to reimburse private facilities at a uniform rate for childbirth, whether it be birth through vaginal or cesarean delivery. Such a policy would provide financial incentives to encourage vaginal delivery, as has been implemented in Taiwan.^42^

Professional associations in many countries have developed guidelines and recommendations for the prevention of primary cesarean deliveries.^43^ Because no such guidelines exist in India, the Indian Medical Association could be given the responsibility of developing such guidelines. A movement is currently under way in India to ensure that the cesarean delivery rates of all hospitals are made available to the general public with the aim of calling attention to hospitals with high cesarean delivery rates. Some countries have encouraged midwifery-led units as a way to reduce cesarean delivery rates.^43,44^ In addition, for any policy to be successful, cultural factors and local context will need to be considered.^43^

## Conclusions

This cross-sectional study indicates that there is a substantial discrepancy in cesarean delivery rates between the public and private sectors in India, and that private sector health care facilities are associated with increases in cesarean delivery rates. It appears that India is in the early stages of a pattern of increasing cesarean deliveries. Given the context of India, with its expanding middle class, rapidly expanding private sector, low governmental regulatory capacity, and governmental policy that encourages public-private partnerships, conditions seem favorable for the increase in cesarean delivery rates to occur in highly populated states. Hence, it is important that policy makers in India address the public health concern of increasing cesarean deliveries. Further research is needed to understand the factors underlying the substantial increase in cesarean deliveries among private sector health facilities in India.

## Notes

Supplement. **eTable.** Cesarean Deliveries From 1992 to 2016 **eFigure 1.** Percentage of Cesarean Deliveries by Type of Health Facility Among Institutional Births in India, 2005 to 2016 **eFigure 2.** Funnel Plot for Proportion of Cesarean Deliveries in Private Facilities by State and Union Territory in India, 2005 to 2006 **eFigure 3.** Funnel Plot for Proportion of Cesarean Deliveries in Private Facilities by State and Union Territory in India, 2015 to 2016
