# Supplementary material for: Assessment of Variation in Cesarean Delivery Rates Between Public and Private Health Facilities in India From 2005 to 2016
Source: JAMA Netw Open. 2020 Aug 28;3(8):e2015022. doi: 10.1001/jamanetworkopen.2020.15022 (PMC7455857; doi:10.1001/jamanetworkopen.2020.15022)
Supplement: Supplement. — eTable. Cesarean Deliveries From 1992 to 2016 eFigure 1. Percentage of Cesarean Deliveries by Type of Health Facility Among Institutional Births in India, 2005 to 2016 eFigure 2. Funnel Plot for Proportion of Cesarean Deliveries in Private Facilities by State and Union Territory in India, 2005 to 2006 eFigure 3. Funnel Plot for Proportion of Cesarean Deliveries in Private Facilities by State and Union Territory in India, 2015 to 2016 [file jamanetwopen-3-e2015022-s001.pdf]

Supplementary Online Content

Bhatia M, Banerjee K, Dixit P, Dwivedi LK. Assessment of variation in cesarean delivery rates between public and private health facilities in India from 2005 to 2016. *JAMA Netw Open*. 2020;3(8):2015022. doi:10.1001/jamanetworkopen.2020.15022

eTable. Cesarean Deliveries From 1992 to 2016

eFigure 1. Percentage of Cesarean Deliveries by Type of Health Facility Among Institutional Births in India, 2005 to 2016

eFigure 2. Funnel Plot for Proportion of Cesarean Deliveries in Private Facilities by State and Union Territory in India, 2005 to 2006

eFigure 3. Funnel Plot for Proportion of Cesarean Deliveries in Private Facilities by State and Union Territory in India, 2015 to 2016

This supplementary material has been provided by the authors to give readers additional information about their work.

© 2020 Bhatia M et al. *JAMA Network Open*.

eTable. Cesarean Deliveries From 1992 to 2016

States	NFHS 1 (1992-93)			NFHS 2 (1998-99)			NFHS 3 (2005-06)			NFHS 4 (2015-16)		
	Public	Private	CSD	Public	Private	CSD	Public	Private	CSD	Public	Private	CSD
Andaman and Nicobar Islands	N.A.	N.A.	N.A.	N.A.	N.A.	N.A.	N.A.	N.A.	N.A.	16.9	86.2	19.3
Andhra Pradesh	8.3	15.1	4.4	17.4	31.5	14.5	21.5	42.6	22.2	25.5	57.0	40.1
Arunachal Pradesh	4.2	20.0	1.0	13.2	25.9	5.4	10.5	15.3	3.0	12.5	37.5	8.9
Assam	9.2	13.7	1.4	20.8	22.0	3.8	21.3	31.1	5.3	12.9	53.3	13.4
Bihar	4.4	10.8	1.0	21.1	18.7	3.0	7.6	17.3	3.1	2.6	31.0	6.2
Chandigarh	N.A.	N.A.	N.A.	N.A.	N.A.	N.A.	N.A.	N.A.	N.A.	19.5	44.0	22.6
Chhattisgarh	N.A.	N.A.	N.A.	N.A.	N.A.	N.A.	24.9	34.2	4.1	5.7	46.6	9.9
Dadra and Nagar Haveli	N.A.	N.A.	N.A.	N.A.	N.A.	N.A.	N.A.	N.A.	N.A.	12.0	38.4	16.2
Daman and Diu	N.A.	N.A.	N.A.	N.A.	N.A.	N.A.	N.A.	N.A.	N.A.	7.3	26.8	15.8
Delhi	8.9	11.8	4.6	20.8	24.3	13.5	18.1	29.0	13.8	26.5	41.5	26.7
Goa	11.3	20.1	13.7	11.5	30.5	20.2	18.0	37.2	25.8	19.9	51.3	31.4
Gujarat	2.0	11.1	2.7	10.0	20.5	8.5	13.8	17.8	8.9	10.8	26.6	18.4
Haryana	10.6	13.6	2.0	14.4	20.4	4.3	14.4	15.3	5.3	8.6	25.3	11.7
Himachal Pradesh	7.6	4.7	1.2	14.2	31.9	5.1	26.4	48.3	12.6	16.4	44.4	16.7
Jammu and Kashmir	18.4	25.2	4.4	23.6	38.2	9.3	24.9	38.2	13.5	35.1	75.5	33.1
Jharkhand	N.A.	N.A.	N.A.	N.A.	N.A.	N.A.	15.9	22.1	3.9	4.6	39.5	9.9
Karnataka	7.1	13.3	3.7	16.3	27.9	11.1	17.3	33.4	15.5	16.9	40.3	23.6
Kerala	11.8	17.6	13.2	28.0	33.8	29.3	26.0	32.7	30.1	31.4	38.6	35.8
Lakshadweep	N.A.	N.A.	N.A.	N.A.	N.A.	N.A.	N.A.	N.A.	N.A.	27.1	59.9	38.4
Madhya Pradesh	1.0	1.3	0.8	9.4	27.1	3.3	6.8	29.0	3.5	5.8	40.8	8.6
Maharashtra	4.7	10.7	3.4	11.8	16.7	7.7	11.7	22.2	11.6	13.1	33.1	20.1
Manipur	0.8	0.0	0.2	14.5	17.4	5.5	17.7	27.0	9.0	22.6	46.2	21.1
Meghalaya	7.7	10.9	2.6	13.4	20.0	2.9	8.3	27.9	4.1	9.8	31.4	7.6
Mizoram	5.9	4.8	2.8	16.9	22.8	10.8	8.9	19.0	6.2	12.3	30.1	12.7
Nagaland	8.6	0.0	1.3	15.2	12.1	1.7	15.8	19.7	2.0	13.4	31.5	5.8
Odisha	6.6	11.7	1.2	19.5	26.2	5.0	10.0	32.8	5.1	11.5	53.7	13.8
Puducherry	N.A.	N.A.	N.A.	N.A.	N.A.	N.A.	N.A.	N.A.	N.A.	30.4	48.3	33.6
Punjab	18.9	14.9	4.2	28.0	18.7	8.2	34.3	31.8	16.6	17.8	39.7	24.6
Rajasthan	5.1	3.2	0.7	11.5	13.6	3.1	11.7	14.7	3.8	6.1	23.2	8.6
Sikkim	N.A.	N.A.	N.A.	22.6	35.9	7.3	25.0	44.3	12.3	18.1	49.3	20.9
Tamil Nadu	9.9	12.1	7.1	13.2	25.6	15.8	14.9	33.2	20.3	26.3	51.3	34.1
Telangana	N.A.	N.A.	N.A.	N.A.	N.A.	N.A.	N.A.	N.A.	N.A.	40.3	74.5	57.7
Tripura	10.6	0.0	3.3	14.7	52.9	8.0	23.7	69.5	12.9	18.1	73.7	20.5
Uttar Pradesh	5.7	5.8	0.7	11.8	22.3	2.8	11.1	25.9	4.4	4.7	31.3	9.4
Uttarakhand	N.A.	N.A.	N.A.	N.A.	N.A.	N.A.	22.3	28.0	8.1	9.3	36.4	13.1
West Bengal	10.1	24.0	4.0	20.8	40.1	10.3	16.6	47.6	10.2	18.8	70.9	23.8
**India**	**7.2**	**12.3**	**2.5**	**15.7**	**24.6**	**7.1**	**15.2**	**27.9**	**8.5**	**11.9**	**40.9**	**17.2**

Note: N.A. Data not available or not collected
CSD: Caesarean Section Deliveries out of total births
Chhattisgarh, Jharkhand and Uttarakhand were carved out of Madhya Pradesh, Bihar and Uttar Pradesh, respectively, in 2000. Andhra Pradesh was divided into Andhra Pradesh and Telangana in 2014.

© 2020 Bhatia M et al. *JAMA Network Open*.

eFigure 1. Percentage of Cesarean Deliveries by Type of Health Facility Among Institutional Births in India, 2005 to 2016

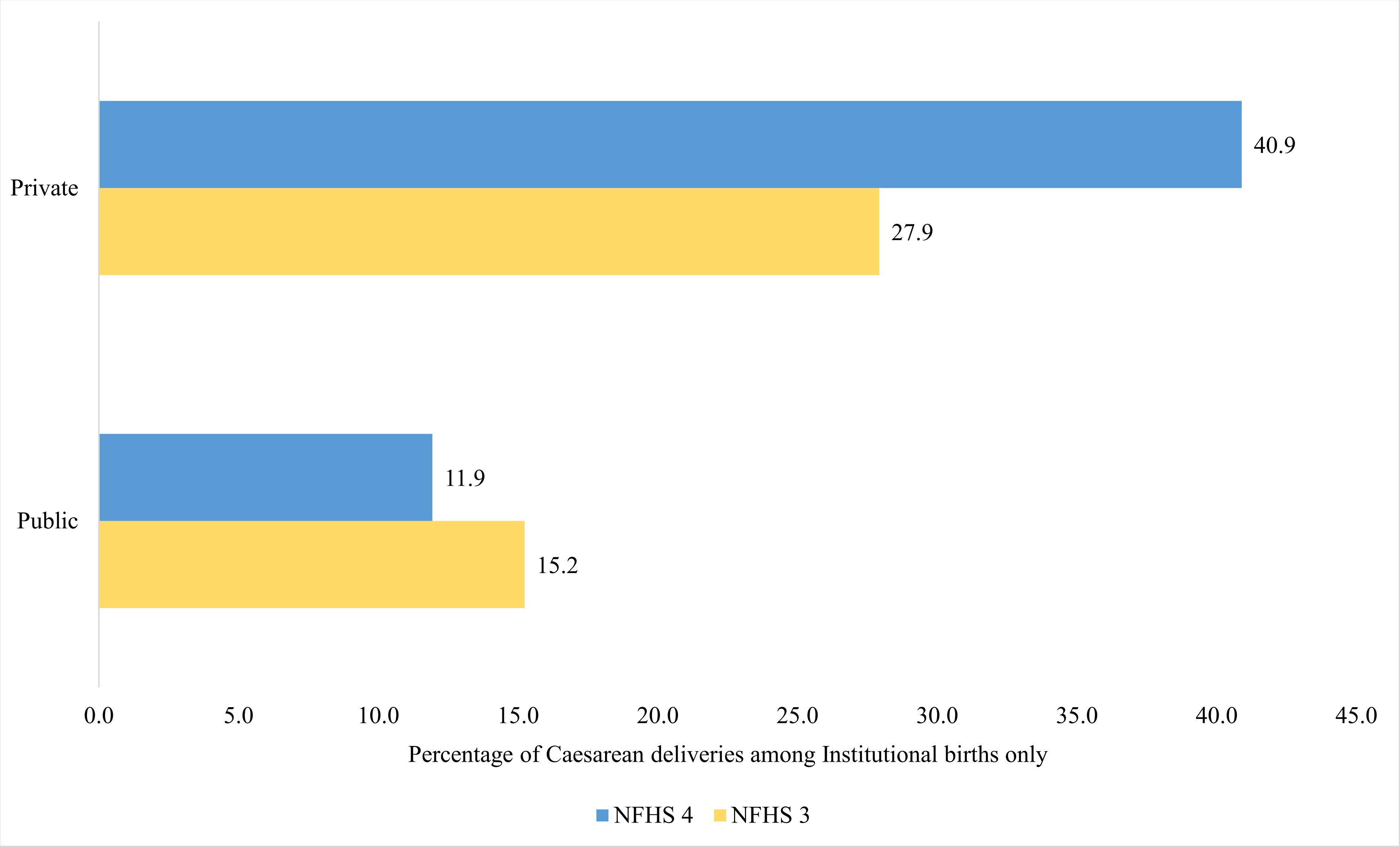

© 2020 Bhatia M et al. *JAMA Network Open*.

eFigure 2. Funnel Plot for Proportion of Cesarean Deliveries in Private Facilities by State and Union Territory in India, 2005 to 2006

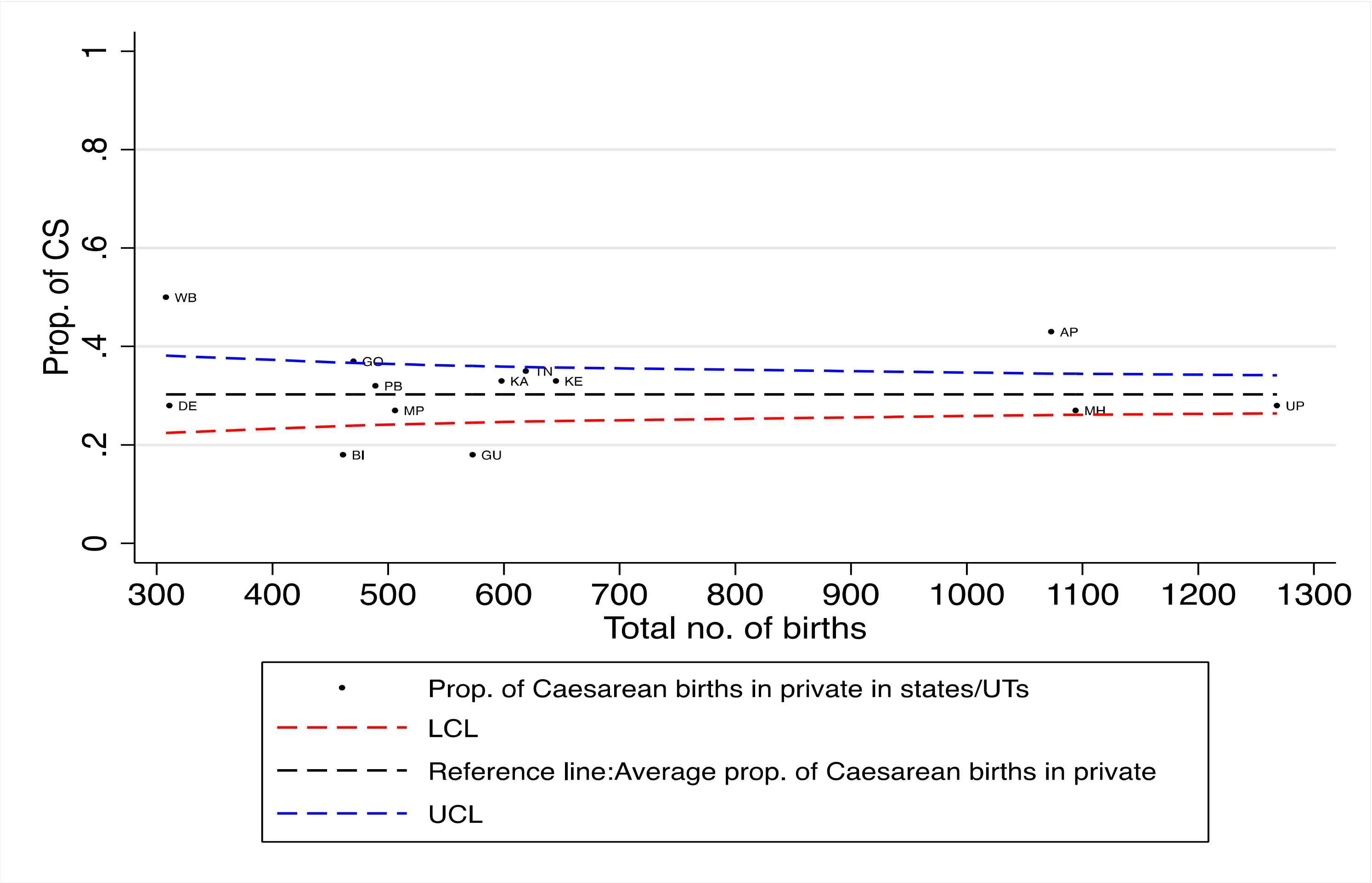

© 2020 Bhatia M et al. *JAMA Network Open*.

eFigure 3. Funnel Plot for Proportion of Cesarean Deliveries in Private Facilities by State and Union Territory in India, 2015 to 2016

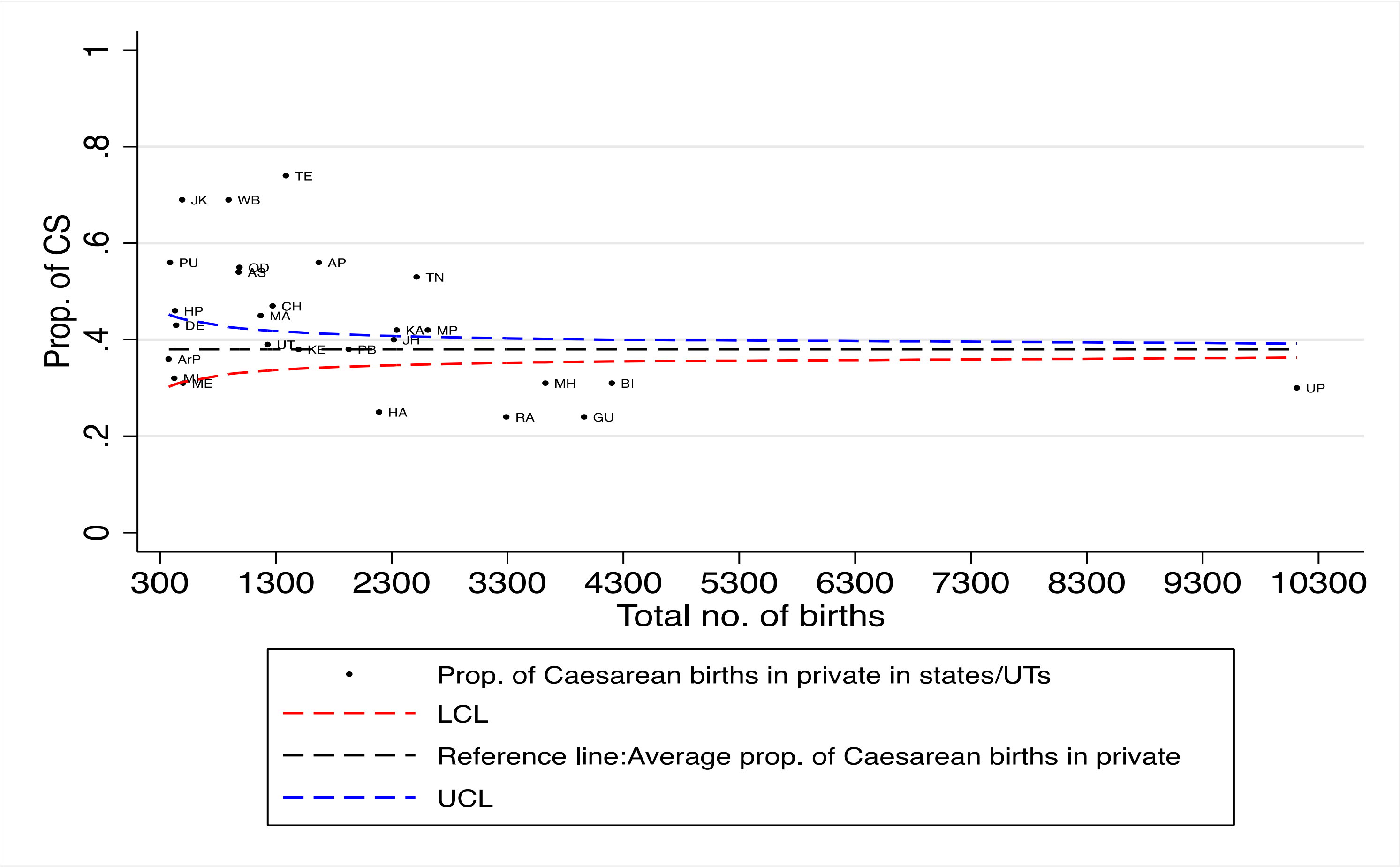

Note: Estimates of unweighted proportion of caesarean deliveries in private facilities by states and UTs along with 95% confidence intervals are provided in eFigure 2 and eFigure 3.

Only those states/UTs with more than 300 recorded sampled births in private facilities are shown in these graphs.

CS-Caesarean Section, LCL-Lower Control Limit, UCL- Upper Control Limit.

The abbreviations of States and Union Territories are:

Andaman and Nicobar Islands (AN), Andhra Pradesh (AP), Arunachal Pradesh (ArP), Assam (AS), Bihar (BI), Chandigarh (CG), Chhattisgarh (CH), Dadra and Nagar Haveli (DN), Daman and Diu (DD), Delhi (DE), Goa (GO), Gujarat (GU), Haryana (HA), Himachal Pradesh (HP), Jammu and Kashmir (JK), Jharkhand (JH), Karnataka (KA), Kerala (KE), Lakshadweep (LD), Madhya Pradesh (MP), Maharashtra (MH), Manipur (MA), Meghalaya (ME), Mizoram (MI), Nagaland (NA), Odisha (OD), Puducherry (PD), Punjab (PU), Rajasthan (RA), Sikkim (SI), Tamil Nadu (TN), Telagana (TE), Tripura (TP), Uttar Pradesh (UP), Uttarakhand (UT), West Bengal (WB)

© 2020 Bhatia M et al. *JAMA Network Open*.
